# Supplementary figures and images for: A genomic variant of ALPK2 is associated with increased liver fibrosis risk in HIV/HCV coinfected women
Source: PLoS One. 2021 Mar 11;16(3):e0247277. doi: 10.1371/journal.pone.0247277 (PMC7951908; doi:10.1371/journal.pone.0247277)

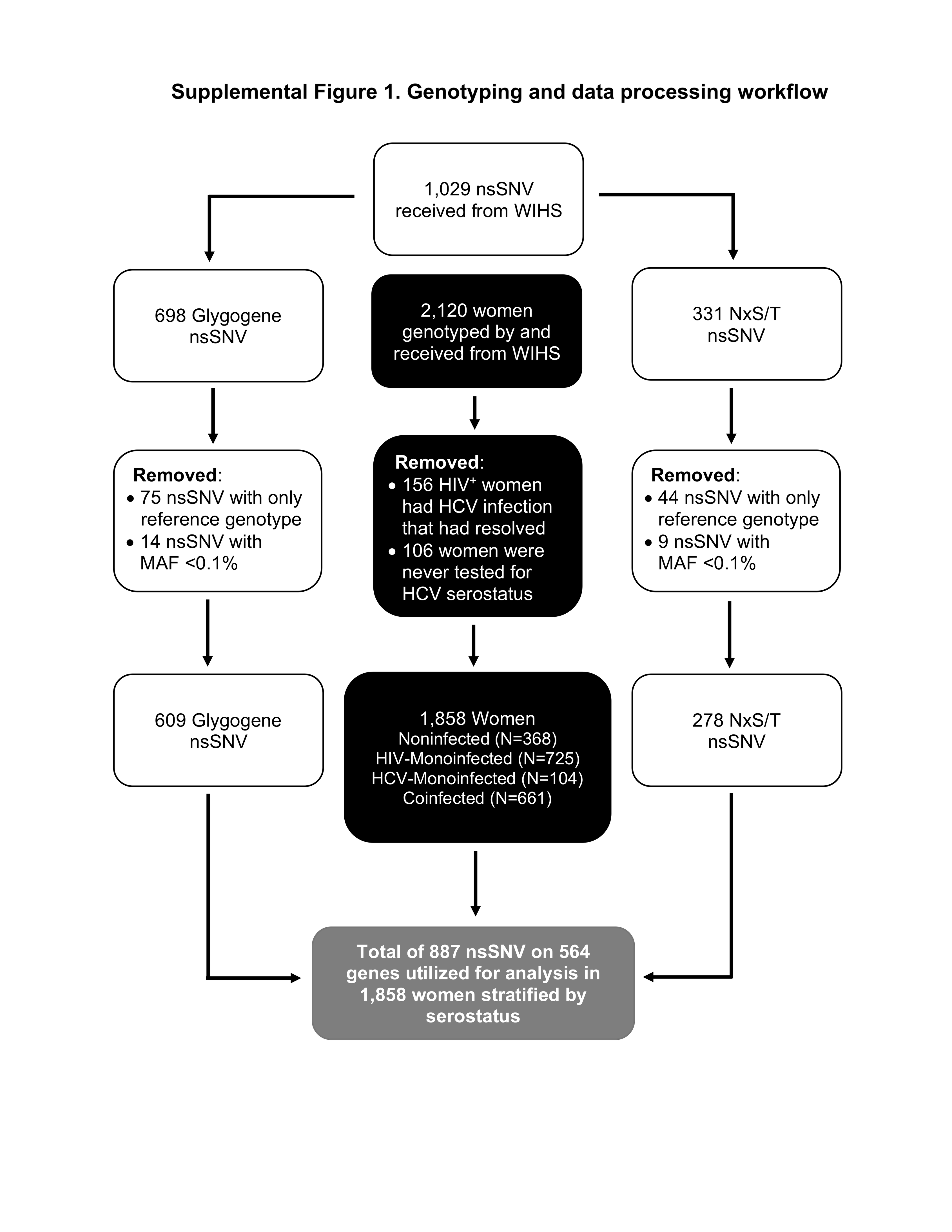

Supplement: S1 Fig — (TIF) [file pone.0247277.s001.tif]

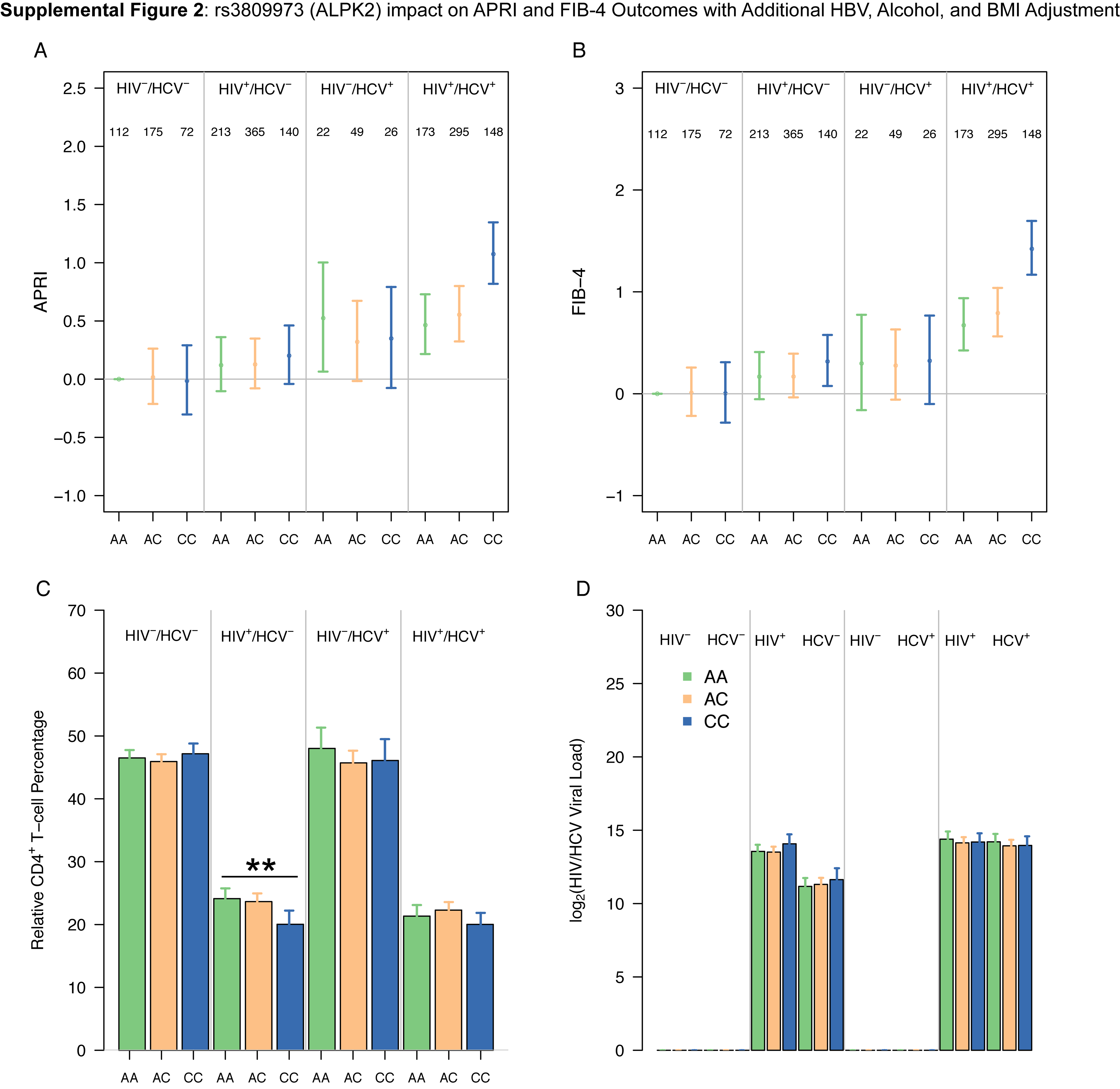

Supplement: S2 Fig — Mean baseline APRI (A) and FIB-4 (B) score with 95% confidence interval shown for the allele pairs of each serotype (Total N = 1,790). The number of samples representing each genotype for the respective serogroups is displayed as follows: homozygous for the major allele (green), heterozygous (orange), and homozygous for the minor allele (blue). Comparisons were shown, for reference, relative to the major allele homozygote of the noninfected serogroup. Different from Fig 3, Outcome was additionally adjusted for factors influencing liver fibrosis including HBV infection status, Alcohol usage, and BMI. ANOVA was used to compare relative CD4+ T-cell percentages (C) or HIV/HCV viral loads (D) between the genotypes of each serogroup. Asterisks indicate two-sided P-values below 0.05 (*) and 0.01 (**) respectively. (TIF) [file pone.0247277.s002.tif]

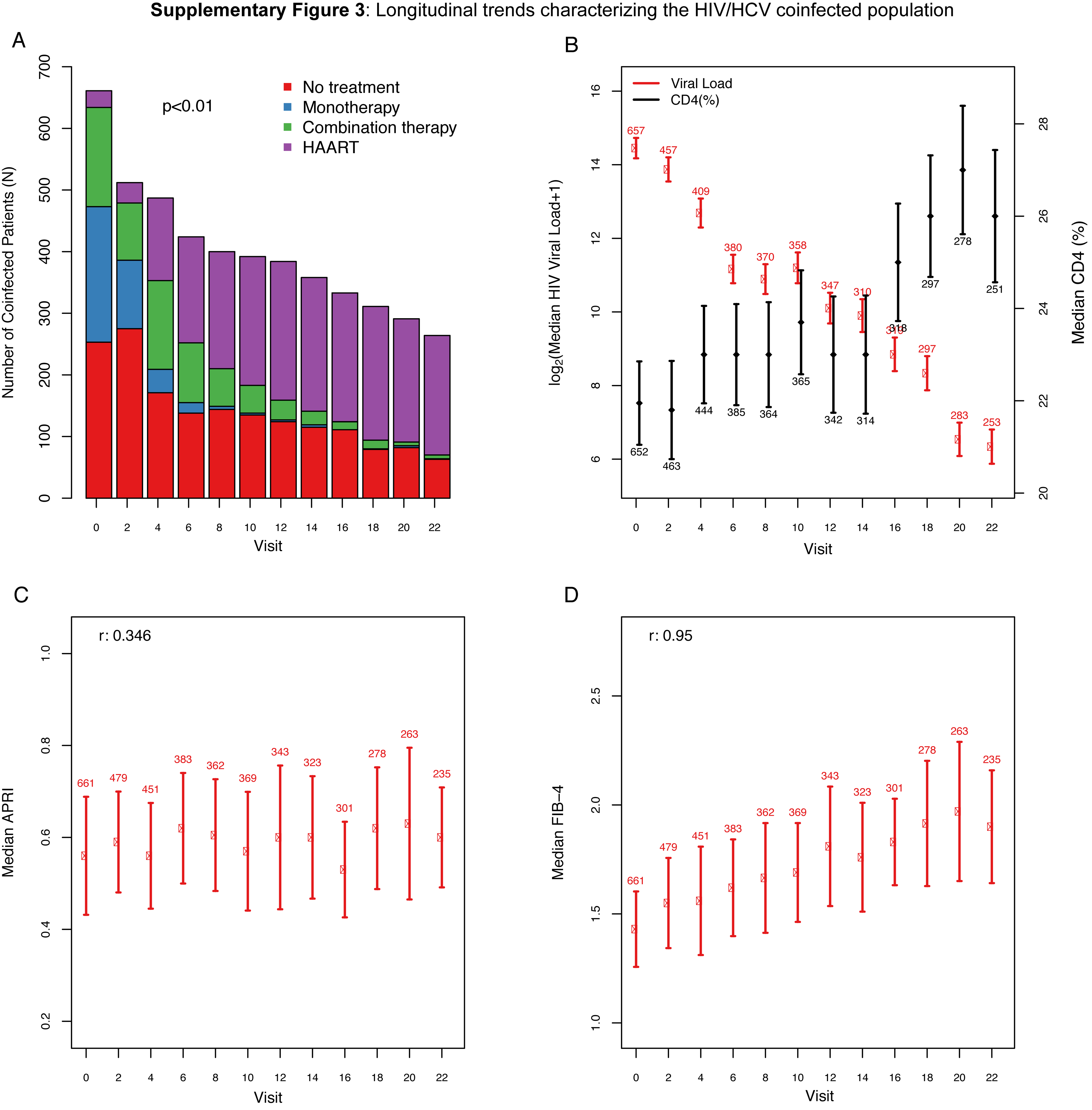

Supplement: S3 Fig — The values are summarized annually at every second visit. (A) Composition of the anti-HIV drug regimens changing in line with treatment guidelines; no therapy (red), monotherapy (blue), combination therapy (green), or highly active antiretroviral therapy (HAART) (purple). (B) Mean CD4+ percentages (black symbols) and mean HIV viral load (red symbols). (C) The median APRI and (D) FIB-4 indexes. (TIF) [file pone.0247277.s003.tif]
