# Supplementary material for: A genomic variant of ALPK2 is associated with increased liver fibrosis risk in HIV/HCV coinfected women
Source: PLoS One. 2021 Mar 11;16(3):e0247277. doi: 10.1371/journal.pone.0247277 (PMC7951908; doi:10.1371/journal.pone.0247277)
Supplement: S3 Table — (DOCX) [file pone.0247277.s006.docx]

| **Supplemental Table 3. Baseline screen of multiple linear regression models with additional adjustment for Alcohol, HBV, and BMI (NAFLD)** | | | | | | | | | |
| --- | --- | --- | --- | --- | --- | --- | --- | --- | --- |
| **Model Fit (N= 616)** | | | **SNV + Age + PC1-3 + HIV Load + HCV Load** **+**  **Alcohol Usage + HBV Status + BMI**^†^ | | | | | | **Study**  **Sample MAF** |
| **Outcome** |  |  | **APRI** | | | **FIB-4** | | |  |
| **SNV** | **Alleles (N)** | | **Estimate** | ***P*** | ***P*_FDR_** | **Estimate** | ***P*** | ***P*_FDR_** |  |
| rs3809973 | CC | (148) | 0.59 | <0.01 | **0.02** | 0.71 | <0.01 | 0.21 | 0.48 |
| *ALPK2* | AC | (295) | 0.11 | 0.36 |  | 0.18 | 0.33 |  |  |
| (K829N)^‡^ | AA | (173) | 0 |  |  | 0 |  |  |  |
| rs3745925  *MADCAM1*  (P300H) | TT  TG  GG | (24)  (191)  (401) | 0.82  -0.04  0 | <0.01  0.72 | 0.38 | 1.57  -0.05  0 | <0.01  0.75 | **0.04** | 0.19 |
| rs2307145  *IL12RB2*  (Q426H) | CC  CG  GG | (4)  (77)  (535) | 2.22  -0.11  0 | <0.01  0.46 | 0.21 | 3.73  -0.17  0 | <0.01  0.46 | **0.04** | 0.07 |
| rs17035120 | TT | (4) | 2.08 | <0.01 | 0.31 | 3.86 | <0.01 | **0.04** | 0.07 |
| *GLT8D2* | TC | (73) | -0.06 | 0.72 |  | 0.14 | 0.56 |  |  |
| (A37T)^‡^ | CC | (539) | 0 |  |  | 0 |  |  |  |
| rs1800450 | AA | (6) | 1.53 | <0.01 | 0.23 | 3.31 | <0.01 | **<0.01** | 0.06 |
| *MBL2* | AG | (58) | -0.31 | 0.09 |  | -0.40 | 0.14 |  |  |
| (G54D) | GG | (552) | 0 |  |  | 0 |  |  |  |
| rs77452813  *MCOLN2*  (V20I) | TT  TC  CC | (2)  (48)  (566) | 3.46  -0.14  0 | <0.01  0.49 | 0.11 | 6.07  -0.17  0 | <0.01  0.56 | **<0.01** | 0.04 |
| rs52828316  *MAN2A2*  (R1039H) | AA  AG  GG | (2)  (35)  (579) | 7.46  -0.29  0 | <0.01  0.18 | **<0.01** | 6.29  -0.46  0 | <0.01  0.17 | **<0.01** | 0.03 |
| rs2228015  *CCR7*  (M7V) | TC  TT | (20)  (596) | 1.01  0 | <0.01 | 0.11 | 1.75  0 | <0.01 | **0.01** | 0.02 |
| rs1800472  *TGFB1*  (T263I) | TC  CC | (11)  (605) | 2.29  0 | <0.01 | **<0.01** | 1.69  0 | <0.01 | 0.29 | 0.01 |
| **Bold** Indicates Significant *P* Values.  ^†^ Multiple Linear Regression of each SNV adjusted for Age (continuous), Race (PC1-PC3), HIV/HCV Load (log2 copies/mL; continuous), Alcohol Use (Abstainers, Mild, Heavy usage stratification), HBV Status (Negative vs. Positive), and BMI (continuous).  Single Letter Amino Acid Abbreviation and Position of Resulting Substitution. | | | | | | | | | |
| ^‡^Potential Gain or Loss of an NxS/T Caused by Substitution.  **Abbreviations:** PC, Principle Component; NxS/T, N-linked Glycosylation Sequon; SNV, Single Nucleotide Variant; MAF, Minor Allele Frequency, FDR, False Discovery Rate; BMI, Body Mass Index. | | | | | | | | | |
